# Endoscopic ultrasound-guided tissue acquisition for splenic lesions: A systematic review and meta-analysis of diagnostic test accuracy

**DOI:** 10.1371/journal.pone.0276529

**Published:** 2022-10-20

**Authors:** Xiao Pan, Shu Huang, Peiling Gan, Lei Shi, Huifang Xia, Xinyi Zeng, Han Zhang, Muhan Lü, Xian Zhou, Xiaowei Tang

**Affiliations:** 1 Department of Gastroenterology, The Affliated Hospital of Southwest Medical University, Luzhou, China; 2 Nuclear Medicine and Molecular Imaging Key Laboratory of Sichuan Province, Luzhou, China; 3 Department of Gastroenterology, the People’s Hospital of Lianshui, Huaian, China; Affiliated Hospital of Nanjing University of Chinese Medicine: Jiangsu Province Academy of Traditional Chinese Medicine, CHINA

## Abstract

**Background and aims:**

At present, it is difficult and risky to diagnose splenic lesions by conventional needle biopsy using computed tomography (CT) or ultrasound (US). Endoscopic ultrasound (EUS)-guided tissue acquisition is increasingly being used as a new technique to determine the tissue diagnosis of splenic lesions. Therefore, our goal was to determine the efficacy and safety of EUS-guided tissue acquisition for splenic lesions.

**Methods:**

We performed a systematic review and meta-analysis to evaluate the pooled sensitivity and specificity of EUS-guided tissue acquisition for the diagnosis of splenic lesions using Metadisc. The Quality Assessment of Diagnostic Accuracy Studies Questionnaire, a quality assessment tool, was used to scrutinize the quality of the studies.

**Results:**

Six eligible studies between January 2000 and June 2022 were identified, and a total number of 62 patients (aged range from 19 to 84) were enrolled. One patient was excluded because of insufficient specimens. The pooled sensitivity and specificity of included studies were 0.85 [95% confidence interval (CI), 0.73–0.93] and 0.77 (95% CI, 0.46–0.95), respectively. The pooled positive likelihood ratio (LR) was 2.38 (95% CI, 1.24–4.57), the pooled negative LR was 0.31 (95% CI, 0.17–0.55), the pooled diagnostic odds ratio (DOR) was 8.67 (95% CI, 2.80–26.82), the area under the summary receiver operating characteristic (SROC) curve was 0.8100 (Standard Error 0.0813).

**Conclusion:**

EUS-guided tissue acquisition is a safe technique with high sensitivity in the diagnosis of splenic lesions. However, because of the small sample sizes, more studies with more cases are needed to further validate these results.

## Introduction

Splenic lesions are seldom encountered in clinical practice, and it is detected incidentally on abdominal-computed tomography (CT), ultrasound (US), or magnetic resonance imaging (MRI) [1]. At present, it is difficult and risky to diagnose splenic lesions by conventional needle biopsy guided by CT or US [2]. The spleen is a hematopoietic organ surrounded by vital structures, such as the lung, kidney, and colon, limiting access and increasing the dangers of percutaneous tissue acquisition [3]. The emergence of Endoscopic ultrasound (EUS)-guided tissue acquisition has changed this situation, which takes advantage of the proximity of the gastric wall to the spleen to puncture and visualize the needle as well as its movements in real-time. EUS-guided tissue acquisition is increasingly being used as a new technique to determine the tissue diagnosis of splenic lesions [4–6]. Lisotti et al. have estimated the pooled adequacy (92.8%) and accuracy (88.2%) of EUS-guided tissue aspiration of splenic lesions [7]. However, no quantitative and comprehensive diagnostic test accuracy (DTA) review has been conducted to determine the sensitivity, specificity, and predictive value of these diagnostic tests. Therefore, we performed a systematic review and DTA meta-analysis to better estimate the diagnostic efficiency of EUS-guided tissue acquisition for splenic lesions.

## Methods

We carried out and reported this systematic review and meta-analysis based on the Preferred Reporting Items for Systematic Reviews and Meta-analysis of Diagnostic Test Accuracy Studies (PRISMA-DTA) [8,9].

### Literature search

We performed a comprehensive literature search in PubMed, Web of Science, Embase, and the Cochrane Library until June 2022. The search terms were ((splenic lesions) OR (splenic)) AND ((endoscopic ultrasound guided biopsy) OR (EUS-guided biopsy) OR (EUS-guided fine needle aspiration) OR (EUS-guided FNA) OR (Endosonography)). Two researchers screened the title and abstract independently according to inclusion and exclusion criteria and compared the results. The disagreement will be resolved through discussion with a third reviewer.

### Inclusion and exclusion criteria

Studies met following criteria were included: 1) reported the diagnostic performance of EUS-guided biopsy 2) 2*2 table: true positive (TP), false positive (FP), false negative (FN), and true negative (TN) rates can be acquired from studies directly or indirectly, 3) The final diagnosis came from the result of EUS-FNA cytology, patient follow up or final surgical pathology diagnosis, 4) published in English, 5) the number of included patients was 5 or more. Studies without final diagnosis, case report, duplicated literature, animal studies, laboratory studies, not published in English and reviews were excluded.

### Data selection and extraction

We constructed a characteristics list to extract the pertinent information about authors, country, year of publication, the number of male and female, and the median patient age, sample size, number of needle passes, type of needle for EUS, splenic lesion size and adverse events. According to extracted data, we constructed a 2*2 table for each study with the number of true-positive, false-positive, true-negative, and false-negative results.

### Quality assessment

QUADAS-2 (Quality Assessment of Diagnostic Accuracy Studies) was used to assess the quality of the selected studies [10]. This questionnaire was composed of 4 domains: patient selection, index test, reference standard, flow and timing. Based on QUADAS-2 tool, each artical were assessed in terms of the risk of bias and concerns about applicability, and they were rated as yes, no, or unclear.

### Statistical methods

According to extracted data, we constructed a 2*2 contingency table for each study, and 0.5 was added to all cells in the table. The pooled sensitivity, specificity, positive likelihood ratio (LR), negative LR, diagnostic odds ratio (DOR) with 95% confidence interval (CI) for the diagnostic accuracy of EUS-FNA in splenic lesions, and summary receiver operating characteristic (SROC) curve were analyzed using software Meta-Disc, version 1.4 (Ramona Cajal Hospital, Madrid, Spain). The I^2^ statistic was used to quantify the heterogeneity between studies. If I^2^ ≥ 50% or Q-test p≤0 .10, DerSimonian-Laird random-effect model was applied. Instead, Mantel-Haenzsel fixed-effects model was used. A useful statistic in pooling studies by means of the ROC curve is the area under the curve (AUC) which summarizes the diagnostic performance as a single number: a perfect test will have an AUC close to 1, and poor tests have AUCs close to 0.5. Then, the heterogeneity resulted from threshold effect was explored by sROC and Spearman coefficient.

## Result

### Description of the included studies

**Fig 1:**
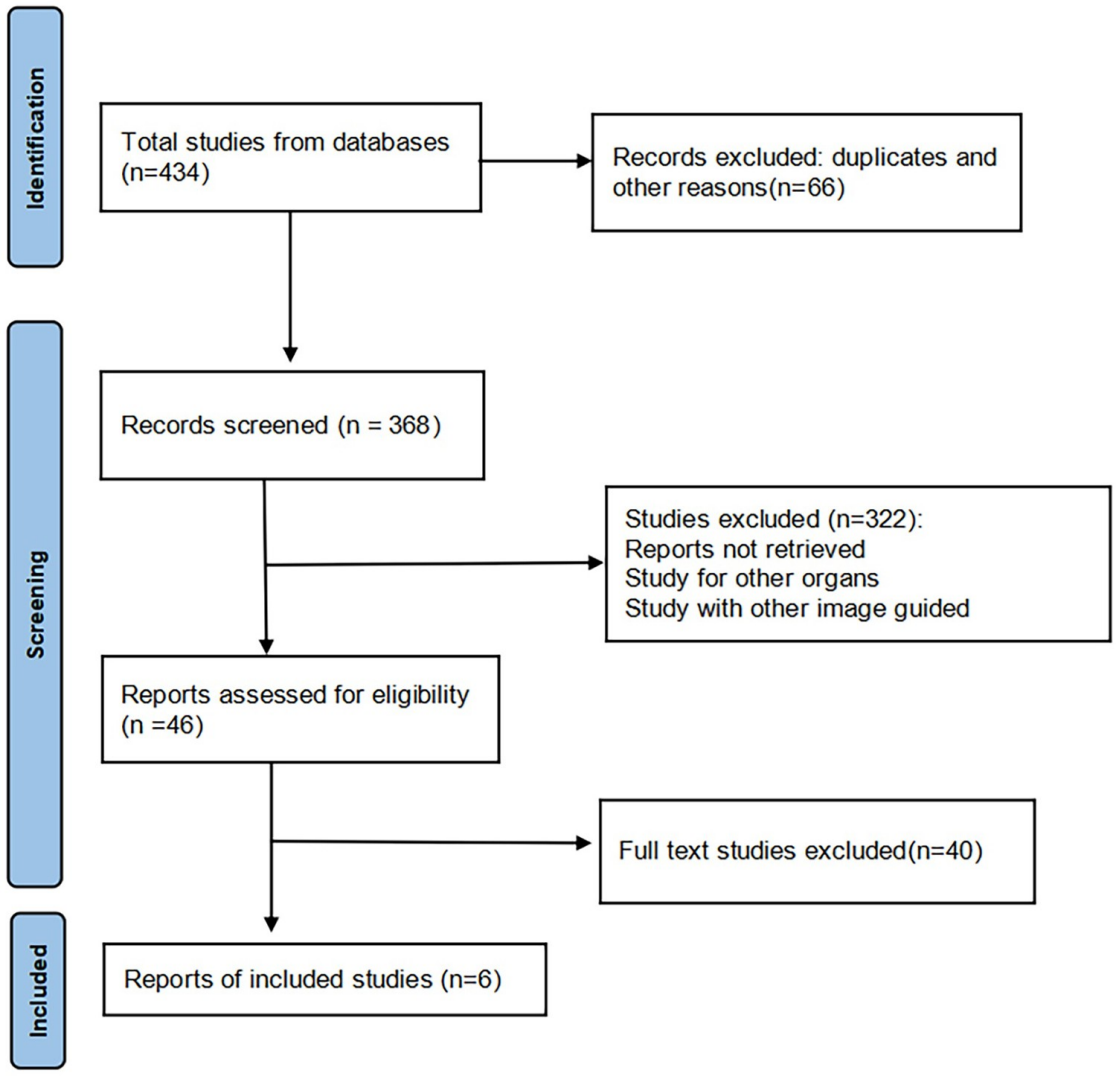
Flow diagram for identifying eligible studies included in the meta-analysis.

A total of 434 records were found in the initial search: 234 through PubMed, 2 through Cochrane Library, 127 through Embase and 71 through Web of Science. After removing 66 articles for duplicates and other reasons, the title and abstract of 368 papers were reviewed. Through careful screening the title and abstract, 322 articles were excluded, and 40 articles underwent full-text review. Finally, 6 studies were included in quantitative synthesis [11–16]. Fig 1 and S1 File displayed a flow diagram of literature search. And the PRISMA checklist was shown in S1 Checklist.

### Study characteristics

The original information extracted from each included studies were recorded on S2 File to better manage and analyze data. The detailed characteristics were summarized in Table 1. These studies were conducted between 2003 and 2021. The median age of patients ranged from 32 to 67. All of included studies used FNA (Fine Needle Aspiration) needles. Several kinds of needles were used. Some studies (n = 8) used a 19-G or 22-G, or 25-G needle, some (n = 3) used a combination of two or three needle sizes. One patient developed septic shock due to the splenic abscess 10 days after the procedure. In addition, two patients were reported mild pain after endoscopic ultrasound-guided biopsy, and one patient had a massive hemorrhage from a splenic artery pseudoaneurysm 7 days after FNA. The EUS-FNA procedures of four included studies were performed by experienced endosonologist [11,12,13,15]. Another two studies didn’t mention the experience of their performers.

**Table 1:** Characteristics of included articles.

Author	Country	Year	Median age (years)	No.of patients	F/M	Needle type	Needle used	Median passes	No.with insufficient specimens	Median size of splenic lesion (mm)	No.of adverse events
Annette et al.	United Kingdom and Germany	2003	32	12	5/7	FNA	22G (n = 12)	3	1	long axis 14	1
Gabriel et al.	Spain	2020	67	15	9/6	FNA	19G (n = 1), 22G (n = 15)	3	0	25*35	0
Eloubeidi et al.	America	2006	58.5	6	2/4	FNA	22G (N = 6)	4.5	0	25*26	0
Surinder et al.	India	2017	35.5	16	5/11	FNA	19G (n = 1), 22G (n = 13), 25G (n = 2)	1.5	0	long axis 15	1
Iwashita et al.	Japan	2009	67	5	4/1	FNA	19G (n = 5)	2	0	45*53	1
Niiya et al.	Japan	2021	68.5	8	2/6	FNA	22G (n = 5), 25G (n = 3)	NA	0	NA	0

F/M: Female/male; NA: Not available.

### Quality of studies

**Fig 2:**
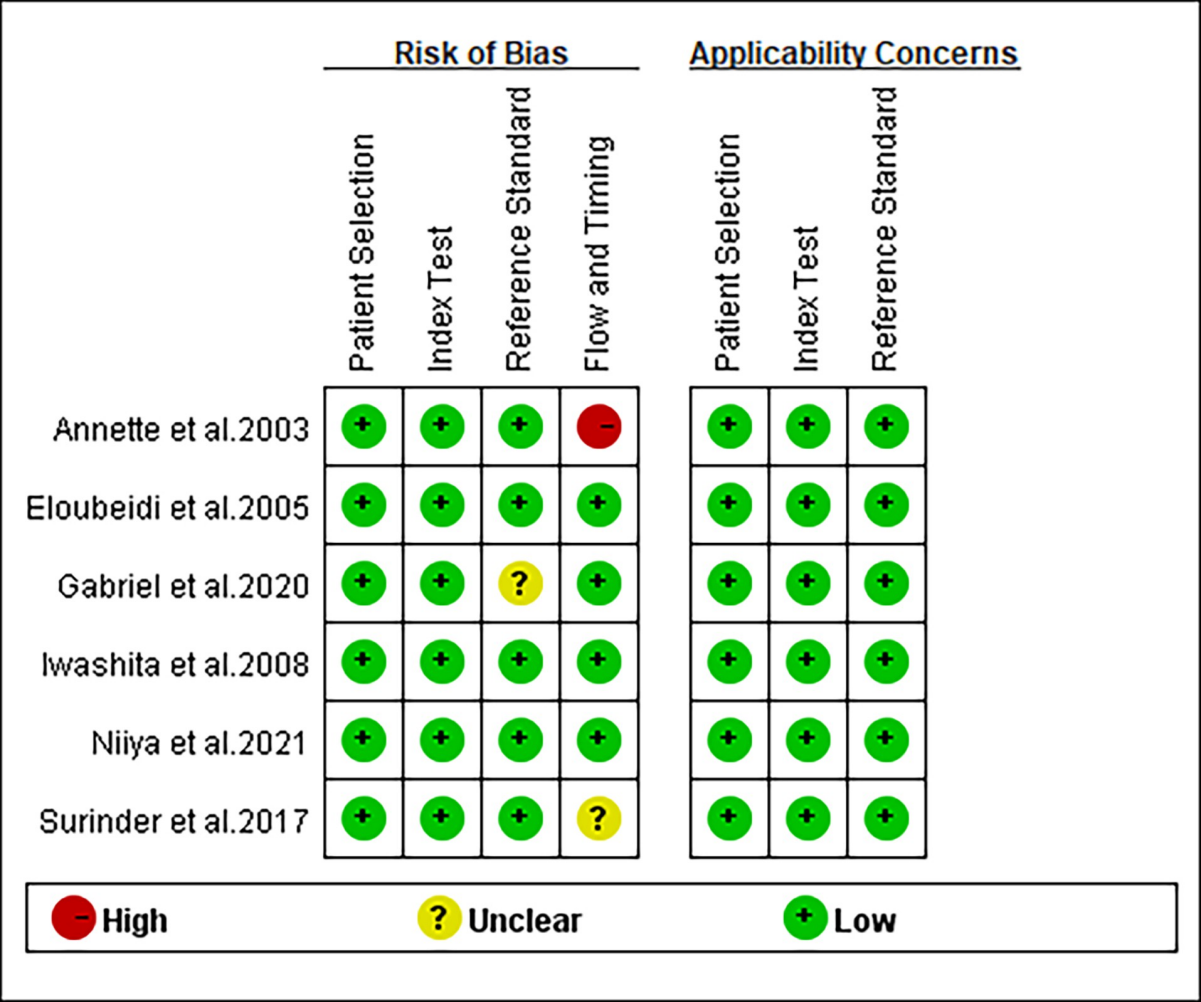
Quality assessment of the included studies by Quality Assessment of Diagnostic Accuracy Studies questionnaire. Green indicates absence of bias, red indicates the presence of bias, and yellow indicates unclear.

The quality of the included studies evaluated using the QUADAS-2 was shown in Fig 2. In general, these studies were scored as high quality.

In the study by Annette et al. [11], the risk of bias in the flowing and timing was scored as “high risk” because one patient was not included in the analysis because of inadequate cytology. In another study [12], the risk of bias in the flow and timing was scored as “unclear risk” for lacking the interval of follow-up. The risk of bias in the reference standard of the study of Gabriel et al. [15] were scored as “unclear risk” for lacking the diagnostic criteria of final diagnosis.

### Yield of EUS for splenic leisons

**Fig 3:**
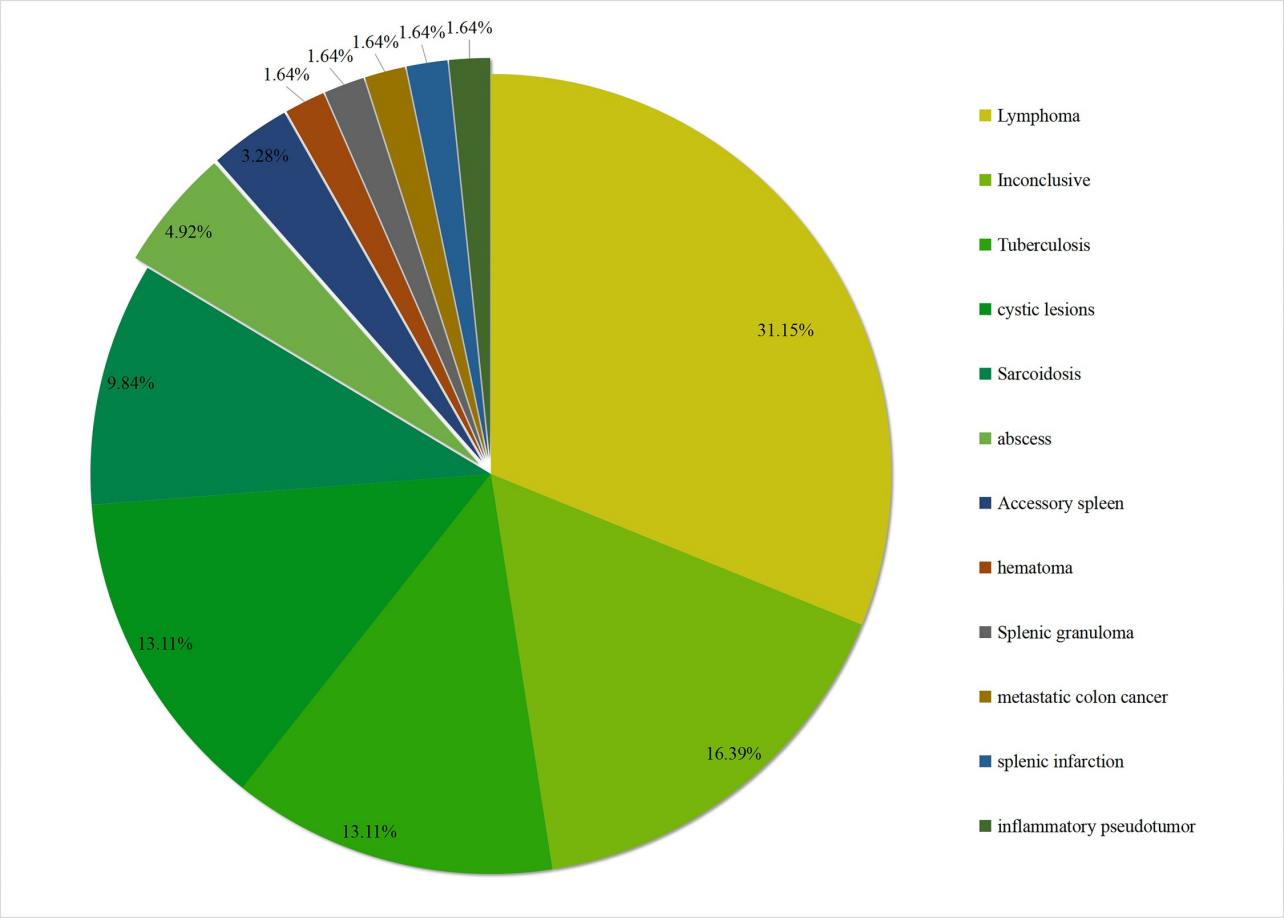
Cytologic diagnoses of endoscopic ultrasound-guided splenic lesions biopsy.

The cytologic diagnoses of splenic lesions is shown in Fig 3. Amongst 61 cases, there were 19(31.15%) lymphoma, 8(13.11%) tuberculosis, 8 (13.11%) cystic lesions, 6 (9.84%) sarcoidosis, 3 (4.92%) abscess, 2 (3.28%) accessory spleen. And another 5 patients were diagnosed with hematoma, splenic granuloma, metastatic colon cancer, splenic infarction, inflammatory pseudotumor respectively. An inconclusive diagnosis was achieved by EUS-guided biopsy in 10 patients (16.39%).

### Meta-analysis

**Fig 4:**
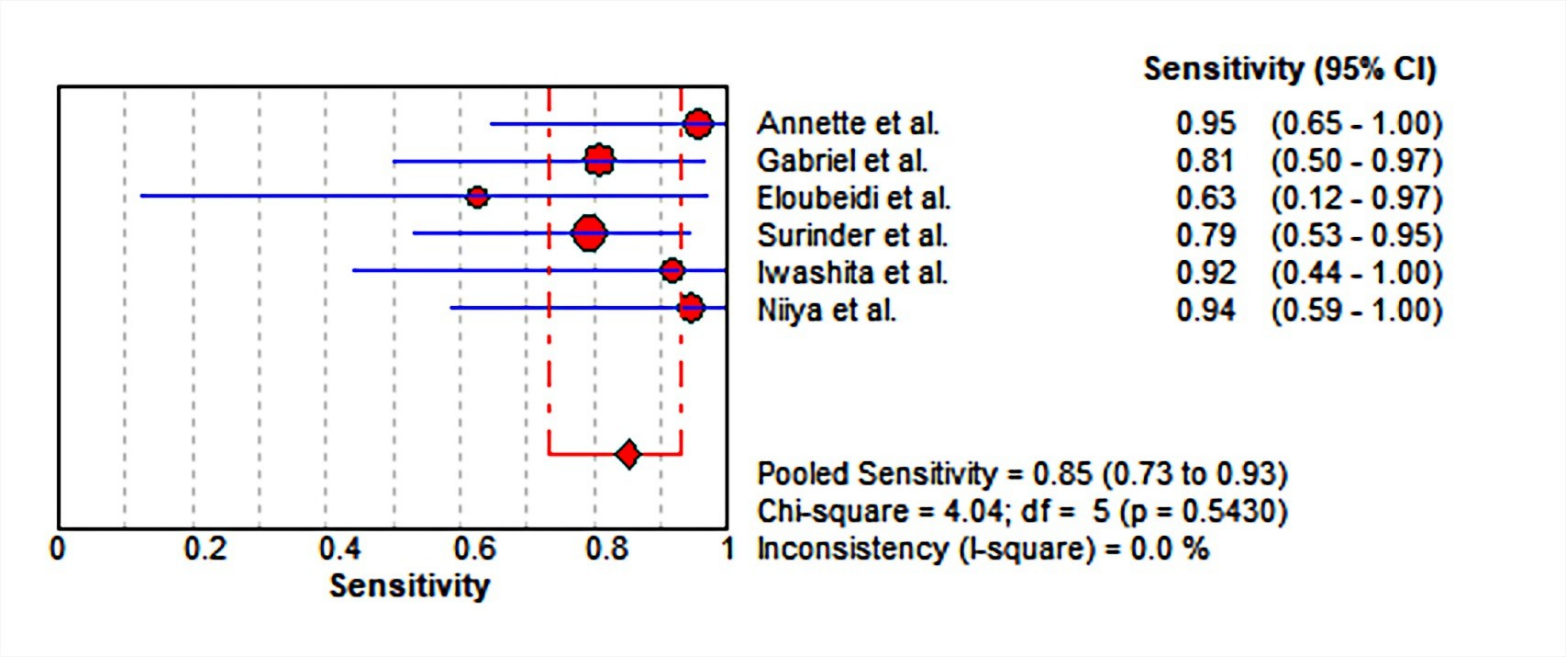
Forest plot showing pooled sensitivity for EUS-guided biopsy for the diagnosis of splenic lesions. CI, confidence interval; df, degrees of freedom.

**Fig 5:**
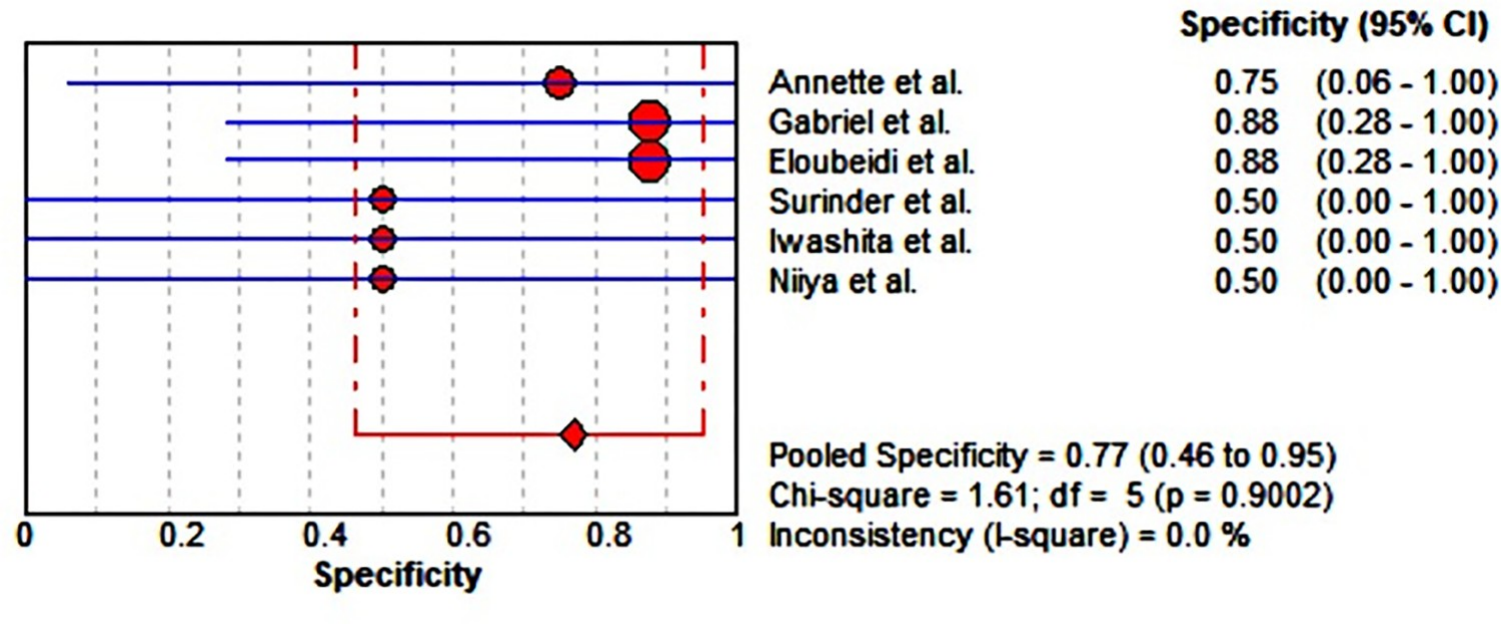
Forest plot showing pooled specificity for EUS-guided biopsy for the diagnosis of splenic lesions. CI, confidence interval; df, degrees of freedom.

**Fig 6:**
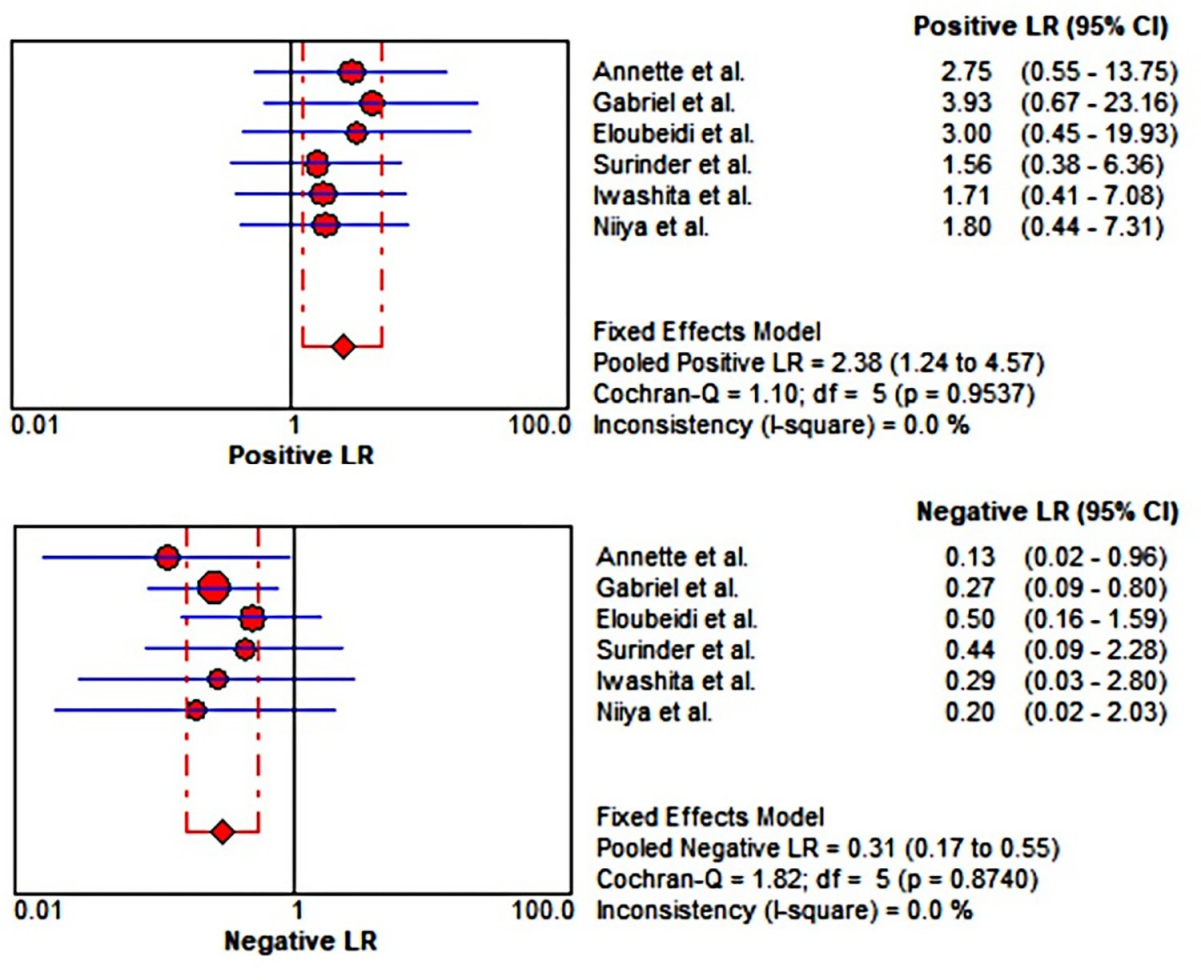
Forest plot showing positive LR and negative LR for EUS-guided biopsy for the diagnosis of splenic lesions. LR, likelihood ratio.

**Fig 7:**
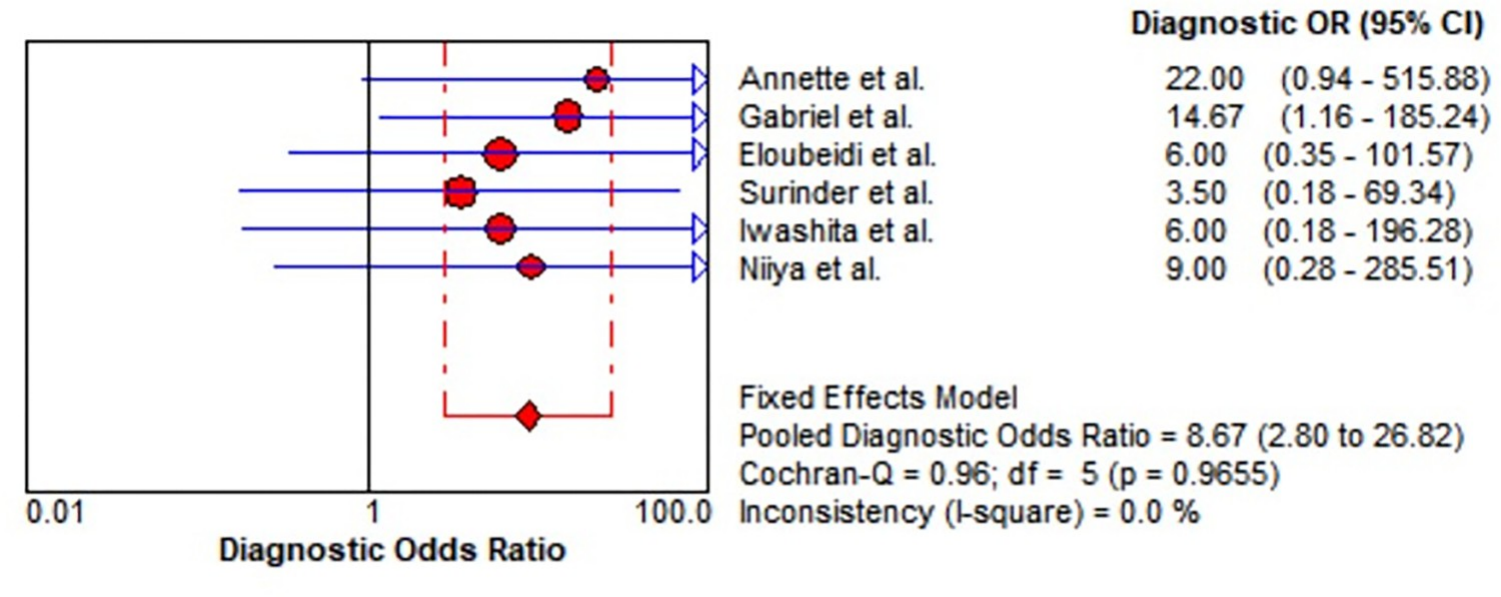
Forest plot showing diagnostic odds ratio of EUS-guided biopsy for the diagnosis of splenic lesions.

**Fig 8:**
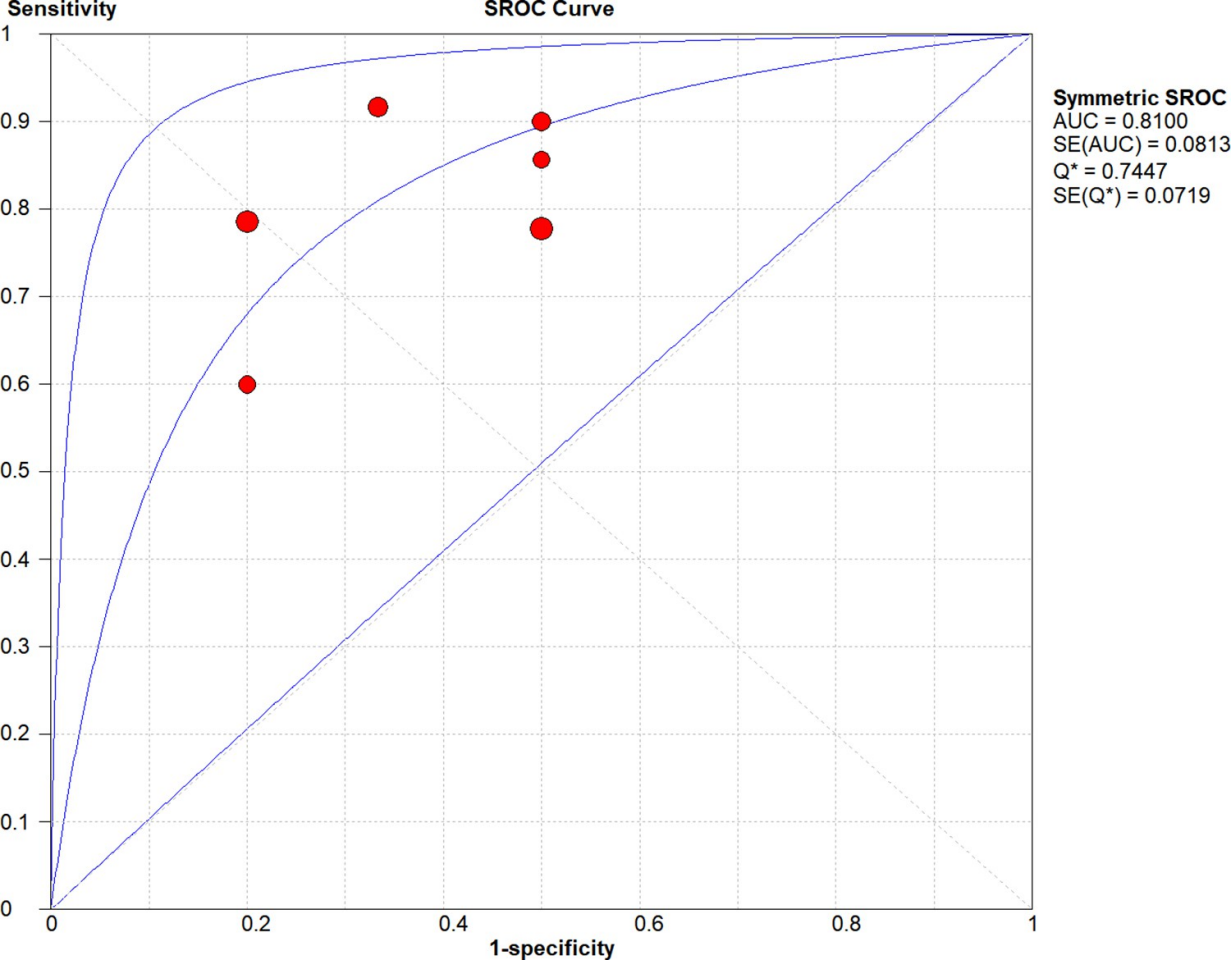
The summary receiver operating characteristic curve of EUS-guided biopsy for the diagnosis of splenic lesions. sROC, summary receiver operating characteristic; AUC, area under curve; SE, standard error.

Six studies including 61 lesions were assessed for the diagnostic performance of EUS-guided tissue acquisition for the diagnosis of splenic lesions. The 2*2 table of diagnostic parameters from included studies was shown in Table 2. There were no heterogeneity (all I^2^ = 50%), therefore Mantel-Haenszel fixed-effects model was utilized to perform the analysis. The pooled sensitivity and specificity were 0.85 (95% CI, 0.73–0.93) (Fig 4) and 0.77 (95% CI, 0.46–0.95) (Fig 5), respectively. The pooled positive LR was 2.38 (95% CI, 1.24–4.57), the pooled negative LR was 0.31 (95% CI, 0.17–0.55), the pooled DOR was 8.67 (95% CI, 2.80–26.82) (Figs 6 and 7). This result also supported by the visual inspection of the SROC plots and the area under the SROC curve was 0.8100 (Fig 8). The Spearman correlation coefficient was 0.339 (p = 0.510) (S1 Fig).

**Table 2:** The diagnostic parameters of the included studies.

Author	year	True positive	False positive	False negative	True negative
Annette et al.	2003	10	0	0	1
Gabriel et al.	2020	10	0	2	3
Eloubeidi et al.	2005	2	0	1	3
Surinder et al.	2017	13	0	3	0
Iwashita et al.	2008	5	0	0	0
Niiya et al.	2021	8	0	0	0

## Discussion

Since its appearance in the early 1980s, EUS has offered preferable visualization of the structure of gastrointestinal wall and neighboring abdominal organs [17]. EUS-guided tissue acquisition provides a improved means of diagnosis, allowing the advantage of shortening hospital stay and reducing the need for major surgery [18]. Focal lesions in the spleen are rare as compared with those in other solid viscera. Splenic lesions are often incidentally detected on abdominal-CT, ultrasound, or MRI. However, it is difficult to define merely based on clinical and radiologic findings [19]. It was reported that about 12% of splenomegaly admissions from 1963 to 1995 required a diagnostic splenectomy [20]. As far as we know, EUS-guided tissue acquisition has been mentioned in some articles as a good diagnostic method for splenic lesions. EUS-guided tissue acquisition of the spleen is an indication for further management to decide whether perform splenectomy. Therefore, EUS-guided tissue acquisition for diagnosing splenic lesion has gained widespread popularity in the past few years.

In this meta-analysis, we demonstrated that diagnostic capacity of EUS-guided tissue acquisition on splenic lesions performed well, with a pooled sensitivity of 85% and a pooled specificity of 77%, and an area under the sROC curve of 81%. The points in SROC plane didn’t show a curvilinear pattern, and the Spearman coefficient is 0.339 (p = 0.510), both of which mean no threshold effect exists. McInnes et al. [21] assessed the diagnostic accuracy of image (CT, US, or fluoroscopy)-guided percutaneous needle biopsy of the spleen, performing 859 biopsies in 741 patients, with a sensitivity of 87.0% and specificity of 96.4%. It seemed that EUS-guided tissue acquisition had the comparable diagnostic validity. And the low specificity may be likely due to limited data.

In this study, a rare complication of bleeding from a splenic artery pseudoaneurysm was reported. It can thus be estimate that the appearance of vascularity in the splenic lesions might be an important factor of complications. And no other major complication of EUS-guided tissue acquisition was reported. But the incidence of complications for CT/ultrasound-guided tissue acquisition and percutaneous splenic puncture were relatively high, such as bleeding, infection, tumor seeding [22] and an unusual complication-needle fracture [23]. In addition, the risk of major complications following percutaneous splenic biopsy ranged from 1.3% to 5.3% [19,24]. Other typical drawback of percutaneous splenic biopsy is that visibility of spleen may be affected by surrounding environment such as ascites and patients with a history of abdominal surgery. Compared with the data mentioned above, EUS-guided tissue acquisition is an safe technique for the diagnosis of splenic lesions.

There is no agreement on the type of needle used for EUS-guided tissue acquisition for diagnosis of splenic lesions. Sammon et al. [19] considered that fine-needle aspiration cytology has greater security compared with core needle biopsy, but the diagnostic accuracy of core biopsy is higher. However, Amani et al. [25] believed that the combined FNAB/CNB approach is reasonable and a logical technique. Many studies has been published to estimate the performance of different gauge needles. Ramesh et al. [26] conducted a randomized trial of 100 patients accepting EUS-FNA of pancreatic lesions and they found no significant difference between 19G and 25G needles. Similarly, Song et al. [27] reported an observation which compared the diagnostic accuracy of 19G and 22G needles for EUS-FNA of solid pancreatic masses. In their study, the diagnostic accuracy and amount of cellular material obtained of 19G needle was superior than 22G needle. A recent study by Harsh et al. [28] has showed that because specimens appear highly fragmented, thicker needle lead to inferior specimen adequacy. In conclusion, the optimal spleen sampling technique remains unclear. Some studies used EUS-FNA in conjunction with flow cytometry. However, there is a concern that it may not be possible to establish a diagnosis in some splenic lesions by assessment of cytological material alone because of an expectation of a high incidence of clinically unhelpful or equivocal diagnoses. In this analyze, we got a discouraging false-negetive rate of 9.84% (6 of 61 cases). Uma et al. [29] considered false positive rates were not affected by sampling techniques and they were especially higher in lymphoma patients. However, the pooled DOR in our study expressed that the odds of developing disease for the people with a positive results are more than eight times greater than for the people with a negtive results. EUS-guided tissue acquisition provided excellent positive predictive value to diagnose splenic lesions.

The strengths of this review are as follows: Firstly, we carried out rigorous procedure for literature search and data extraction. Secondly, meta-analysis of diagnostic test accuracy was more suitable for included small-sized studies. Tirdly, we discussed that the types of needles had effect on EUS-guided tissue acquisition.

We acknowledged that there are limitations in this study. First, there are more articles which focused only on the diagnosis of splenic lymphoma by EUS-guide tissue acquisition and only a few articles investigated the etiology of all the splenic lesions by EUS-guide tissue acquisition. Our results might be influenced by this situation and change as more statistics are available. Second, we were unable to assesses the impact of different levels of endoscopic expertise, because the accuracy of EUS-guide biopsy in the diagnosis of splenic lesions also depends on the technique of the operators.

In summary, our results demonstrate that EUS-guided tissue acquisition is a safe technique with high sensitivity in the diagnosis of splenic lesions. However, because of the small sample sizes, more studies with more cases are needed to further validate these results.

## Supporting information

S1 Checklist PRISMA checklist. (DOCX)

S1 Fig Analysis of diagnostic threshold of EUS-guided tissue acquisition for the diagnosis of splenic lesions. (TIF)

S1 File PRISMA flow diagram. (DOCX)

S2 File Original data extracted from included studies. (DOCX)
