# Supplementary figures and images for: Endoscopic ultrasound-guided tissue acquisition for splenic lesions: A systematic review and meta-analysis of diagnostic test accuracy
Source: PLoS One. 2022 Oct 20;17(10):e0276529. doi: 10.1371/journal.pone.0276529 (PMC9584539; doi:10.1371/journal.pone.0276529)

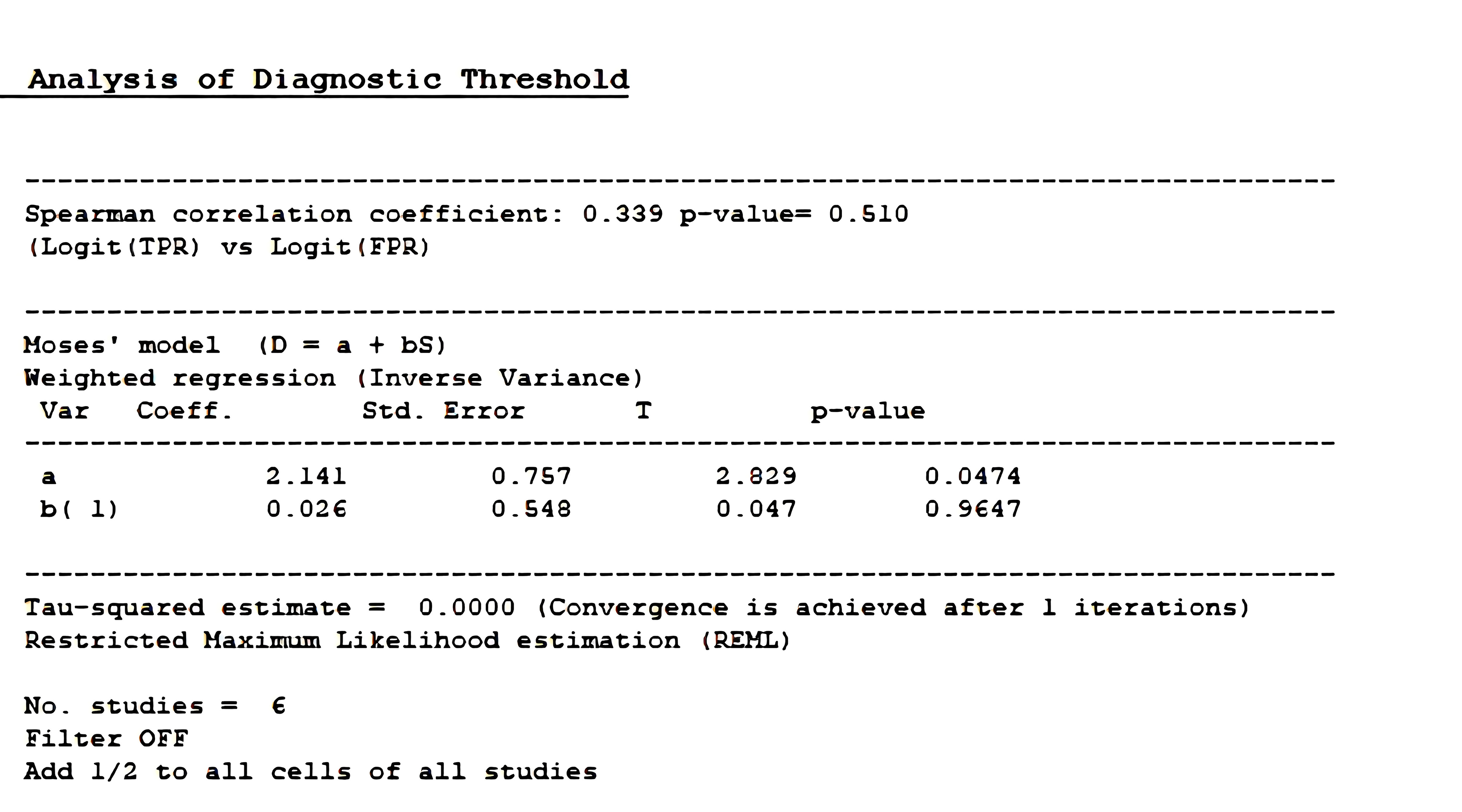

Supplement: S1 Fig — (TIF) [file pone.0276529.s002.tif]
