# Supplementary material for: Endoscopic ultrasound-guided tissue acquisition for splenic lesions: A systematic review and meta-analysis of diagnostic test accuracy
Source: PLoS One. 2022 Oct 20;17(10):e0276529. doi: 10.1371/journal.pone.0276529 (PMC9584539; doi:10.1371/journal.pone.0276529)
Supplement: S1 File — (DOCX) [file pone.0276529.s003.docx]

**Identification**

Records excluded: duplicates and other reasons(n=66)

Total studies from databases (n=434)

Records screened (n = 368)

Studies excluded (n=322):

Reports not retrieved

Study for other organs

Study with other image guided

**Screening**

Reports assessed for eligibility

(n =46)

Full text studies excluded(n=40)

Reports of included studies (n=6)

**Included**

*Consider, if feasible to do so, reporting the number of records identified from each database or register searched (rather than the total number across all databases/registers).

**If automation tools were used, indicate how many records were excluded by a human and how many were excluded by automation tools.

*From:*  Page MJ, McKenzie JE, Bossuyt PM, Boutron I, Hoffmann TC, Mulrow CD, et al. The PRISMA 2020 statement: an updated guideline for reporting systematic reviews. BMJ 2021;372:n71. doi: 10.1136/bmj.n71

For more information, visit: <http://www.prisma-statement.org/>
