# Supplementary material for: Endoscopic ultrasound-guided tissue acquisition for splenic lesions: A systematic review and meta-analysis of diagnostic test accuracy
Source: PLoS One. 2022 Oct 20;17(10):e0276529. doi: 10.1371/journal.pone.0276529 (PMC9584539; doi:10.1371/journal.pone.0276529)
Supplement: S2 File — (DOCX) [file pone.0276529.s004.docx]

| Author & Country | Year | No. | Age (years)  & sex (f/m) | Lesion size | Needle type | Needle used | Needle passes | EUS-FNA diagnosis | | | | Final diagnosis/out come | Complication |
| --- | --- | --- | --- | --- | --- | --- | --- | --- | --- | --- | --- | --- | --- |
|  |  |  |  |  |  |  |  | Cytology | Histology | Bacteriology | Biochemical finding |  |  |
| Annette et al.  United Kingdom and Germany | 2003 | 1 | 19, M | 14 mm | FNA | 22G | 3 | ECG suggestive of tuberculosis | NA | TBC | NA | Tuberculosis | One patient: pain after needle puncture，settled after  4 h. |
|  |  | 2 | 68, M | 10 mm | FNA | 22G | 4 | Benign lymphoid cells | NA | Negative | NA | No signs of recurrent disease |  |
|  |  | 3 | 30, F | 8 mm | FNA | 22G | 3 | ECG suggestive of tuberculosis | NA | TBC | NA | Tuberculosis |  |
|  |  | 4 | 41, M | 28 mm | FNA | 22G | 4 | Hodgkin’s disease | NA | Negative | NA | Recurrent Hodgkin’s disease |  |
|  |  | 5 | 26, F | 14 mm | FNA | 22G | 2 | ECG suggestive of sarcoidosis | NA | Negative | NA | Sarcoidosis |  |
|  |  | 6 | 32, M | 13 mm | FNA | 22G | 3 | Inadequate | NA | Negative | NA | Sarcoidosis |  |
|  |  | 7 | 60, F | 16 mm | FNA | 22G | 2 | Hodgkin’s disease | NA | Negative | NA | Hodgkin’s disease |  |
|  |  | 8 | 72, F | 16 mm | FNA | 22G | 1 | Met colon cancer | NA | No | NA | Surgery met colon cancer |  |
|  |  | 9 | 67, M | 42 mm | FNA | 22G | 2 | Necrosis | NA | Negative | NA | Surgery: infarction |  |
|  |  | 10 | 34, M | 10 mm | FNA | 22G | 2 | Bacteria, leukocytes | NA | *S.* *aureus* | NA | Abscess |  |
|  |  | 11 | 28, M | 14 mm | FNA | 22G | 3 | Leukocytes, abscess | NA | *Serratia* | NA | Abscess |  |
|  |  | 12 | 32, F | 22 mm | FNA | 22G | 3 | ECG suggestive of sarcoidosis | NA | Negative | NA | Sarcoidosis |  |
| Gabriel et al.  Spain | 2020 | 1 | 44, M | ES/25*35 mm | FNA | 22 G | 4 | Inconclusive | | | | NA | None |
|  |  | 2 | 44, F | Normal | FNA | 22 G | 3 | Accessory spleen | | | | NA | None |
|  |  | 3 | 67, M | ES | FNA | 22 G | 3 | Hodgkin lymphoma | | | | NA | None |
|  |  | 4 | 81, F | NS/51*61mm | FNA | 19 G | 1 | hematoma | | | | NA | None |
|  |  | 5 | 56, M | NS/5*7mm | FNA | 22 G | 3 | Splenic granuloma | | | | NA | None |
|  |  | 6 | 86, F | ES | FNA | 22 G | 3 | Non- Hodgkin lymphoma | | | | NA | None |
|  |  | 7 | 81, F | ES/34*54 mm | FNA | 22 G | 2 | Non- Hodgkin Lymphoma | | | | NA | None |
|  |  | 8 | 54, M | ES/14*23 mm | FNA | 22 G | 2 | Splenic abscess | | | | Splenectomy | None |
|  |  | 9 | 70, F | NS | FNA | 22 G | 3 | Inconclusive | | | | NA | None |
|  |  | 10 | 71, M | NS/20*23 mm | FNA | 22 G | 2 | Inconclusive | | | | NA | None |
|  |  | 11 | 65, V | NS/16*21 mm | FNA | 22 G | 2 | Accessory spleen | | | | NA | None |
|  |  | 12 | 58, F | NS/40*45 mm | FNA | 22 G | 3 | Inconclusive | | | | NA | None |
|  |  | 13 | 57, F | ES/50*60 mm | FNA | 22 G | 3 | Non- Hodgkin lymphoma | | | | NA | None |
|  |  | 14 | 70, F | ES/61*72 mm | FNA | 22 G | 3 | Indeterminate | | | | Splenectmy | None |
|  |  | 15 | 84, F | ES/28*37mm | FNA | 22 G | 3 | Non-Hodgkin Lymphoma | | | | NA | None |
| Eloubeidi et al.  America | 2006 | 1 | 54, F | 25*26mm | FNA | 22G | 5 | Lymphoproliferative  disorder | | | | Non-Hodgkin’s  lymphoma | None |
|  |  | 2 | 82, M | Not measured | FNA | 22G | 4 | Lymphoproliferative  disorder | | | | Large B-cell lymphoma | None |
|  |  | 3 | 78, M | 60*70mm | FNA | 22G | 5 | Benign aspirates of  mixed lymphocytes | | | | Large B-cell lymphoma;  confirmed by surgery  False-negative EUS-FNA | None |
|  |  | 4 | 41, F | 17*21mm | FNA | 22G | 4 | Benign aspirate | | | | Benign; confirmed  by surgery | None |
|  |  | 5 | 55, M | 67*89mm | FNA | 22G | 5 | Benign aspirate | | | | Benign; confirmed by  long-term follow-up | None |
|  |  | 6 | 62, M | 20*22mm | FNA | 22G | 4 | Benign aspirate | | | | Benign; confirmed by  long-term follow-up | None |
| Surinder et al.  India | 2017 | 1 | 39, M | Largest 1.5cm | FNA | 22G | 2 | Granuloma | | | | Tuberculosis | Two  patients complained of mild pain in left upper abdomen. |
|  |  | 2 | 32, M | Largest 0.8cm | FNA | 22G | 2 | Granuloma | | | | Sarcoidosis |  |
|  |  | 3 | 28, F | Largest 5.0cm | FNA | 22G | 1 | Non-diagnostic | | | | Simple cyst |  |
|  |  | 4 | 38, F | Largest 1.4cm | FNA | 22G | 2 | Granuloma, acid-fast bacilli+ | | | | Tuberculosis |  |
|  |  | 5 | 35, M | Largest 5.0cm | FNA | 22G | 1 | Elevated amylase and lipase | | | | Pseudocyst |  |
|  |  | 6 | 28, M | Largest 0.6cm | FNA | 22G | 2 | Granuloma | | | | Sarcoidosis |  |
|  |  | 7 | 34, F | Largest 1.2cm | FNA | 22G | 2 | Granuloma | | | | Tuberculosis |  |
|  |  | 8 | 42, M | Largest 1.0cm | FNA | 22G | 2 | Granuloma, acid-fast bacilli+ | | | | Tuberculosis |  |
|  |  | 9 | 32, M | Largest 9.0cm | FNA | 19G | 1 | Elevated amylase and lipase | | | | Pseudocyst |  |
|  |  | 10 | 42, M | Largest 10.0cm | FNA | 22G | 1 | Elevated amylase and lipase | | | | Pseudocyst |  |
|  |  | 11 | 34, M | Largest 6.0cm | FNA | 22G | 1 | Elevated amylase and lipase | | | | Pseudocyst |  |
|  |  | 12 | 46, M | Largest 4.0cm | FNA | 22G | 1 | Elevated amylase and lipase | | | | Pseudocyst |  |
|  |  | 13 | 28, M | Largest 1.2cm | FNA | 25G | 2 | Granuloma, acid-fast bacilli+ | | | | Tuberculosis |  |
|  |  | 14 | 32, F | Largest 1.4cm | FNA | 22G | 1 | Non-diagnostic | | | | Simple cyst |  |
|  |  | 15 | 43, M | Largest 1.2cm | FNA | 25G | 2 | Granuloma | | | | Tuberculosis |  |
|  |  | 16 | 36, F | Largest 4.0cm | FNA | 22G | 1 | Non-diagnostic | | | | Simple cyst |  |
| Iwashita et al.  Japan | 2009 | 1 | 50, F | Max  . 22*13mm | FNA | 19G | 2  (median) | Granuloma | non−caseating  granuloma | Diagnosis: sarcoidosis | | Sarcoidosis | Mild abdominal |
|  |  | 2 | 67, F | 70*51mm | FNA | 19G |  | No atypical  cell | inflammatory pseudotumor | Diagnosis: inflammatory pseudotumor | | Inflammatory pseudotumor | None |
|  |  | 3 | 69, F | Max. 66*46mm | FNA | 19G |  | Lymphoma  suspected. | diffuse large  B−cell lymphoma | Diagnosis: diffuse large B−cell lymphoma | | Diffuse large  B-cell lymphoma | None |
|  |  | 4 | 71, F | Max 14*11mm | FNA | 19G |  | Granuloma | non−caseating  granuloma | Diagnosis: sarcoidosis | | Sarcoidosis | None |
|  |  | 5 | 67, M | 53*45mm | FNA | 19G |  | No atypical  cell | Lymphoma | Diagnosis: lymphoma | | Lymphoma | None |
| Niiya et al.  Japan | 2021 | 1 | 68, F | NA | FNA | 22G | NA | Low-grade B-cell lymphoma | | | | Low-grade B- cell lymphoma | None |
|  |  | 2 | 69, M | NA | FNA | 22G | NA | diffuse large B−cell lymphoma | | | | diffuse large  B−cell lymphoma | None |
|  |  | 3 | 77, M | NA | FNA | 22G | NA | Low-grade B-cell lymphoma | | | | Low-grade B-cell lymphoma | None |
|  |  | 4 | 51, F | NA | FNA | 22G | NA | Low-grade B-cell lymphoma | | | | Low-grade B-cell lymphoma | None |
|  |  | 5 | 79, M | NA | FNA | 25G | NA | Bcell Lymphoma | | | | B-cell lymphoma | None |
|  |  | 6 | 66, M | NA | FNA | 22G | NA | Low-grade B-cell lymphoma | | | | Low-grade B-cell lymphoma | None |
|  |  | 7 | 70, M | NA | FNA | 25G | NA | Low-grade B-cell lymphoma | | | | Low-grade B-cell lymphoma | None |
|  |  | 8 | 55, M | NA | FNA | 25G | NA | B-cell lymphoma | | | | B-cell lymphoma | None |

ECG: epitheloid cell granuloma; F/M: female/male; NA: not available; FNA: fine needle aspiration; ES: enlarged spleen; NS: normal size
